# Treatment-related survival associations of claudin-2 expression in fibroblasts of colorectal cancer

**DOI:** 10.1007/s00428-017-2263-3

**Published:** 2017-11-13

**Authors:** Artur Mezheyeuski, Carina Strell, Ina Hrynchyk, Tormod Kyrre Guren, Anca Dragomir, Tatyana Doroshenko, Oksana Pashkova, Julia Gorgun, Kseniya Ruksha, Per Pfeiffer, Elin H. Kure, Halfdan Sorbye, David Edler, Anna Martling, Bengt Glimelius, Arne Östman, Anna Portyanko

**Affiliations:** 1 Department of Oncology-Pathology, Karolinska Institutet, Cancer Centre Karolinska, R8:03, Stockholm, Sweden; 2 Department of Pathology, Belarusian State Medical University, Minsk, Belarus; 3 City Clinical Pathologoanatomic Bureau, Minsk, Belarus; 4 Department of Oncology, Oslo University Hospital, Oslo, Norway; 5 K.G.Jebsen Colorectal Cancer Research Centre, Oslo University Hospital, Oslo, Norway; 6 Department of Surgical Pathology, Uppsala University Hospital, Uppsala, Sweden; 7 Laboratory of Antibodies and Cytokines Biotechnology, The Republic Research & Production Centre for Transfusiology and Medical Biotechnologies, Minsk, Belarus; 8 Department of Gastroenterology and Nutrition, Belarusian Medical Academy of Postgraduate Education, Minsk, Belarus; 9 N.N. Alexandrov National Cancer Centre of Belarus, Minsk, Belarus; 10 Department of Oncology, University of Southern Denmark, Odense, Denmark; 11 Department of Cancer Genetics, Institute for Cancer Research, Oslo University Hospital, Oslo, Norway; 12 Department of Oncology, Haukeland University Hospital, Bergen, Norway; 13 Department of Molecular Medicine and Surgery, Karolinska University Hospital Solna, 171 76 Stockholm, Sweden; 14 Department of Immunology, Genetics and Pathology, Section of Oncology, Uppsala University, Uppsala, Sweden

**Keywords:** Colorectal cancer, Cell adhesion, Claudin-2, Cancer-associated fibroblasts

## Abstract

Claudin-2 is a trans-membrane protein—component of tight junctions in epithelial cells. Elevated claudin-2 expression has been reported in colorectal cancer (CRC). The aim of this study was to investigate the expression patterns of claudin-2 in human CRC samples and analyze its association with clinical characteristics and treatment outcome. TMAs of primary tumors from two cohorts of metastatic CRC (mCRC) were used. Claudin-2 IHC staining was evaluated in a semi-quantitative manner in different regions and cell types. Claudin-2 expression was also analyzed by immunofluorescence in primary cultures of human CRC cancer-associated fibroblasts (CAFs). Initial analyses identified previously unrecognized expression patterns of claudin-2 in CAFs of human CRC. Claudin-2 expression in CAFs of the invasive margin was associated with shorter progression-free survival. Subgroup analyses demonstrated that the survival associations occurred among cases that received 5-FU+oxaliplatin combination treatment, but not in patients receiving 5-FU±irinotecan. The finding was validated by analyses of the independent cohort. In summary, previously unreported stromal expression of claudin-2 in CAFs of human CRC was detected together with significant association between high claudin-2 expression in CAFs and shorter survival in 5-FU+oxaliplatin-treated mCRC patients.

**Electronic supplementary material:**

The online version of this article (10.1007/s00428-017-2263-3) contains supplementary material, which is available to authorized users.

## Introduction

Up to 25% of colorectal cancer (CRC) patients have synchronous distant metastases at the time of diagnosis, and another 20–25% develop metachronous metastases [4, 29]. Surgical resection of the distant metastases, most often located to the liver, remains the only curative treatment leading to 5-year survival varying from 25 to 74% [7, 9]. KRAS and BRAF mutations are independent predictors for survival among patients who undergo liver metastases resection [12, 26]. Medical treatment of metastatic CRC (mCRC) includes combination chemotherapy with or without addition of targeted agents blocking EGFR-receptor signaling or angiogenesis. With the exception of RAS mutations, used as a marker to identify patients not benefiting from EGFR inhibitors, no biomarkers are clinically used for other pharmacological treatments of mCRC.

Tight junctions (TJs) are the most apical cell–cell adhesions in the epithelial cells. The claudin superfamily transmembrane proteins, including claudin-2, are important components of TJs. Under normal conditions, claudin-2 expression is associated with “leaky” epithelia of proximal tubule and in Bowman’s capsule of the nephron [14]. More recently, TJ proteins have been implied in “noncanonical” functions in epithelial and other cell types. Claudin-2 expression was reported, e.g., in osteoblasts [31], differentiated macrophages [28, 30], and endothelial cells of certain locations [5]. In normal gut, claudin-2 was detected in the intestinal crypts [22] and is overexpressed in inflammatory bowel disease (IBD) [6], CRC [13], and other tumor types [23].

Recently, claudin family proteins were also detected in cancer-associated fibroblasts (CAFs) [11]. Functional significances of these findings remain to be elucidated. Mechanistic in vitro studies have indicated effects on differentiation and migration of claudins in stromal cells [10, 11].

CAFs are the most abundant cells in the solid tumor stroma. Emerging studies from model systems and from analyses of clinical samples indicate that CAFs constitute a diverse set of cells, composed of functionally and clinically relevant subsets, which may regulate tumor initiation, growth, progression, and response to treatment [8, 18]. Concerning the impact of CAFs on drug response, multiple mechanisms have been suggested which can broadly be divided into effects on drug exposure/delivery and effects on drug sensitivity [8, 17].

CAFs, and CAF-derived factors, can control drug delivery by affecting the interstitial fluid pressure (IFP). Reduction of IFP by enzymatic ablation of fibroblast-derived hyaluronan reexpanded the vasculature and improved drug delivery to the tumor site [21]. Similarly, usage of different PDGFR-β-antagonists, targeting CAFs, reduced IFP and improved transcapillary transport and tumoral uptake of chemotherapeutic drugs and radioimmunotherapeutic antibodies [2, 19]. Accordingly, PDGFR inhibitors improved therapeutic effects of cytotoxic drugs [20].

Based on these earlier findings, this study has explored the possibility that claudin-2 expression in CAFs is related to outcome in chemotherapy-treated patients with mCRC.

## Materials and methods

A synopsis of the materials and methods is presented here. Full details are provided in the Supplementary Materials and Methods.

The SPCRC cohort—an unselected population diagnosed with nonresectable mCRC patients during 2003–2006 in three Scandinavian counties [24]—and the NORDIC-VII cohort from the randomized study investigated the effects of combining cetuximab with a regimen of bolus 5-flourouracil (5-FU)/folinic acid (FA) and oxaliplatin (FLOX) in first-line therapy of mCRC [27] were used.

Tissue microarrays (TMAs) were made from formalin-fixed and paraffin-embedded tissue blocks of primary tumor. For the current study, TMAs from 274 and 262 patients from the invasive margin and tumor center, respectively, from the SPCRC cohort and from 315 patients of the NORDIC-VII cohort were available for immunohistochemistry.

### Immunohistochemical staining

Single- or double-staining procedures were applied for the TMA material with the antibodies to claudin-2 (Thermo Fisher Scientific Cat# 32-5600), CD68 (clone PG-M1; Dako, Inc., Denmark), and pan-cytokeratin antibody (clone AE1/AE3; Dako, Inc., Denmark). The abundance of the claudin-2 in tumor cells was evaluated as an integrated score, considering intensity of the expression and percentage of positive cells, using a four-graded scale (negative (0), weak (1), moderate (2), or strong (3)) and then dichotomized for survival analysis. Claudin-2-positive macrophage score was based on the quantity of marker-positive macrophages, irrespective of expression intensity. Claudin-2-positive CAF score was based on the abundance of dot-like expression (quantity of dots per area) irrespective of expression intensity. Evaluation of the ICH was performed by two pathologists: IH for SPCRC cohort (blinded to clinical and outcome data of SPCRC cohort) and AM for NORDIC-VII cohort (blinded to clinical and outcome data of NORDIC-VII).

### In situ hybridization procedures

RNAscope® 2.5 HD Reagent Kit-RED (Advanced Cell Diagnostics, Hayward, CA) and a custom-designed RNAscope probe targeting 489–1408 of NM_020384.3 were used to detect CLDN2 transcript.

### Fibroblast isolation from tumor tissue

The tissue samples were collected from five CRC patients and used to separate fibroblasts. The cells were subjected to immunofluorescence analysis with antibody to claudin-2 (Thermo Fisher Scientific Cat# 710221 or Thermo Fisher Scientific Cat# 32-5600), α-SMA (clone 1A4; Dako, Inc., Denmark, at dilution 1:300), or E-cadherin ((24E10) rabbit mAb, Cell Signaling Technology, at dilution 1:300).

### Monocyte isolation and differentiation

Human monocytes were isolated from heparinized blood, obtained from healthy donors (The Republic Research & Production Centre for Transfusiology and Medical Biotechnologies, Minsk, Belarus), differentiated into M0-like cells by adhesion on plastic for 30 min, polarized to M2-like macrophages by incubation with 2 ng/ml M-CSF (PeproTech, Rocky Hill, NJ, USA) and/or 40 ng/ml IL-4 (R&D Systems, Minneapolis, MN, USA) for 6 days and stained with antibodies to claudin-2 (Thermo Fisher Scientific Cat# 710221).

### Statistical analyses

Cox proportional hazards model was used to estimate statistical significance and relative hazards in univariate and multivariate settings. Goodman–Kruskal gamma test was used for the analyses of marker expression in different locations. Mann–Whitney *U* test and ANOVA tests were used for the analyses of associations between marker expression and clinical characteristics. All statistical tests were two-sided, and *p* value < 0.05 was considered statistically significant. Due to multiple tests applied for the survival analysis of claudin-2 expression in the SPSS cohort, the Bonferroni correction for the statistical significance was calculated and *p* value = 0.005 was considered as the threshold.

All statistical analyses were performed using SPSS V20 (SPSS Inc., Chicago, IL).

## Results

Initial analyses were performed on tissue sections from human CRC tissue to characterize the patterns of claudin-2 expression. We observed claudin-2 in multiple cell types, including epithelial cells, endothelial cells, CAFs, and macrophages.

### Claudin-2 is expressed in malignant and endothelial cells

**Fig. 1:**
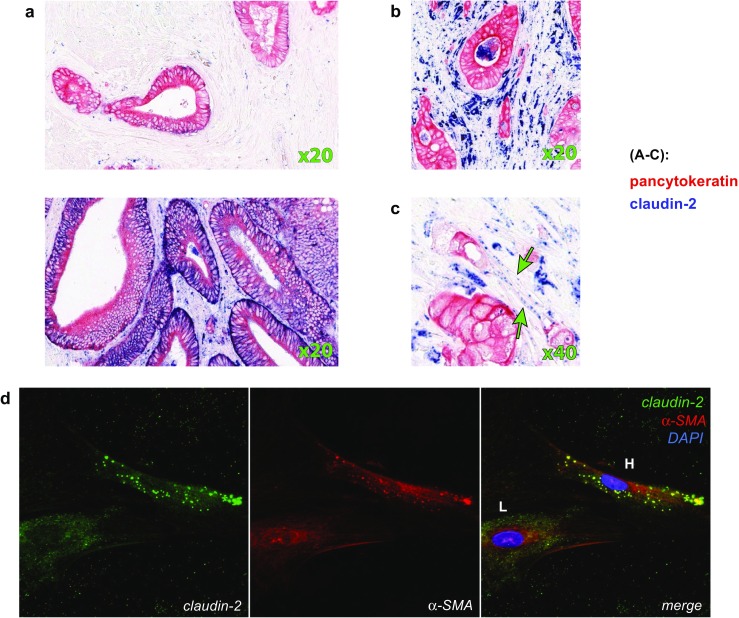
Claudin-2 expression in human colorectal cancer tissue. **a** Representative image of tumor tissue without (upper image) and with high (lower image) claudin-2 expression in cancer cells. Note strong blue staining on pan-cytokeratin (red)-positive areas. **b** Expression of the claudin-2 in macrophages. Note dark-blue spots with irregular shape, frequently with a blank region in the centre (unstained nucleus). **c** Dot-like expression of the claudin-2 in cells with fibroblast-like morphology. **d** Expression of claudin-2, as determined by IF, in a fraction of cells within a primary CAF culture (passage 4). Note claudin-2 dot-like high-level expression in a cell marked “H” and low-level diffuse expression in a cell marked “L” (green) in fraction of CAFs. Red color used for the visualization of α-SMA

In malignant cells, a certain intra- and inter-case variability in the expression levels of claudin-2 was observed (Fig. 1a). Furthermore, different patterns of expression were detected including supra-nuclear, basal, and unpolarized expression (Supp. Fig. 1A).

To investigate the potential impact on survival of claudin-2 expression pattern in malignant cells, an analysis with survival data from the SPCRC cohort was performed. Interesting, an unpolarized pattern was significantly associated with shorter overall survival (OS) (Supp Fig 2) with median 22.8 months for unpolarized pattern, and 38.1 and 38.4 months for supra-nuclear and basal patterns, respectively (*p* = 0.004). No associations with progression-free survival (PFS) were found (data not shown).

We also observed vascular expression of claudin-2, which was associated with endothelial cells (Supp. Fig. 1B). The observation is concordant with earlier reports [25]. A further analysis of vascular expression of claudin-2 was not performed in this study.

### Claudin-2 is expressed in macrophages and CAFs

In agreement with previous studies, the appearance of claudin-2 in macrophages was also demonstrated by the presence of claudin-2/CD68 (pan-macrophage marker) double-positive cells in the stroma (see Fig. 1B and Supp. Fig. 1C). In noncancerous colonic mucosa, the claudin-2-positive macrophages represented a fraction of CD163+ (M2 macrophages) cells with localization restricted to the subepithelial region (see Supp Fig.2A). In the tumor stroma, we observed heterogeneous pattern of claudin-2 expression in macrophages with the presence of CD68+/cl2−, CD68+/cl2+ (see Fig. 1D2), CD163+/cl2− and CD163+/cl2+ cells (see Supp Fig.3B1 and C). Macrophages in peritumoral stroma, not adjacent to the tumor, were predominantly claudin-2-negative (see Supp Fig.3B2).

To extend these findings, we investigated the expression of claudin-2 in human blood monocyte-derived, polarized M2-class macrophages. M2-macrophage-like differentiation of human monocyte cells with M-CSF, IL-4, or M-CSF+IL-4 was reported elsewhere [32] (see “Materials and methods”). M0 cells and IL-4-alone or M-CSF-alone differentiated cells are characterized by low or absent expression of claudin-2 (see Supp Fig. 4A–C and 4E). However, when the combination of M-CSF and IL-4 was used for differentiation, the macrophages expressed high levels of claudin-2 (see Supp Fig. 4D, E).

Apart from the claudin-2-positive macrophages and vessels, additional dispersed dot-like claudin-2 expression was detected in other cells of the tumor stroma (Fig. 1C). These cells displayed an elongated spindle-shaped fibroblast-like morphology and were not juxtaposed to vessels, suggesting that they were CAFs. Additional tests were therefore done to validate the findings from the tissue-based analyses. Cultured primary fibroblasts, derived from surgically resected human CRC, were analyzed by IF. Two subsets of fibroblasts were observed: a subset with low level of cytoplasmic claudin-2 expression (Fig. 1d, “L” marked) and a subset with high level of dot-like claudin-2 expression (Fig. 1d, “H” marked). Both cell subsets were positive to α-SMA (Fig. 1d). CAF-expression of claudin-2 was confirmed in another experiment where cocultures of the primary fibroblasts and the CRC caco-2 cell line revealed claudin-2 expression in the E-cadherin-negative cells (Supp Fig. 5A.).

In order to validate specificity of the IHC staining of the claudin-2, we performed both IHC and in situ hybridization (ISH) on the sequential sections of FFPE sections. The mRNA targeting probes showed a signal pattern, which was similar to IHC expression pattern, with positivity in cancer cells, CAFs, macrophage-like cells, and nonmalignant colon epithelial cells (Supp Fig. 5B.).

### Claudin-2 expression in cancer cells and fibroblasts at the invasive margin of the tumor is associated with KRAS mutation status

Following this initial profiling of claudin-2 expression in human CRC, we focused on the expression of claudin-2 in cancer cells and CAFs. As demonstrated by Goodman–Kruskal gamma test (Supp. Table 1), the expression of claudin-2 in CT and in IM was concordant based on analyses performed separately for cancer cells and CAFs (gamma test g = 0.63 and 0.37, respectively, both *p* < 0.001). When the correlation between the claudin-2 expression in cancer cells and in CAFs of the same location was analyzed, low concordance was observed in both CT and IM (g = 0.28, *p* < 0.001, and g = 0.24, *p* = 0.001, respectively).

To evaluate associations with clinicopathological characteristics, the semiquantitative data of claudin-2 expression was dichotomized into high and low values (see “Materials and methods”). High claudin-2 in malignant cells at the IM was more commonly seen in colon than in rectum tumors. Notably, high claudin-2 expression in malignant cells in the IM was also associated with KRAS, but not BRAF mutation status (Table 1.)

**Table 1:** Associations between claudin-2 expression and clinicopathological parameters in patients with mCRC in the SPCRC cohort

	Tumor center						Invasive margin					
	Cancer cells			Fibroblasts			Cancer cells			Fibroblasts		
	*n* (percent)		*p* value	*n* (percent)		*p* value	*n* (percent)		*p* value	*n* (percent)		*p* value
	Low	High		Low	High		Low	High		Low	High	
Median age (range)	68 (24–96)	71 (26–92)	0.321*	70 (24–96)	71 (28–92)	0.542*	70 (26–96)	71 (24–92)	0.171*	70 (43–96)	71 (24–92)	0.701*
WHO PS												
0	50 (11)	109 (24)		107 (24)	51 (12)		72 (20)	54 (15)		48 (11)	100 (24)	
1	38 (9)	97 (22)	0.812	96 (22)	41 (9)	0.461	45 (13)	55 (16)	0.039	51 (12)	79 (19)	0.189
2–4	43 (10)	105 (24)		93 (21)	54 (12)		77 (22)	48 (14)		42 (10)	103 (24)	
Alk phosph												
Normal	57 (15)	119 (30)	0.430	124 (31)	51 (13)	0.183	72 (23)	62 (20)	0.288	63 (17)	101 (27)	0.139
Elevated	62 (16)	154 (39)		140 (36)	77 (20)		104 (34)	70 (23)		64 (17)	142 (39)	
Gender												
M	72 (16)	146 (33)	0.124	150 (34)	68 (15)	0.417	103 (29)	70 (20)	0.113	77 (18)	128 (30)	0.074
F	59 (13)	165 (38)		146 (33)	78 (18)		91 (26)	87 (25)		64 (15)	154 (37)	
Location												
Colon	93 (21)	238 (55)	0.204	218 (50)	114 (26)	0.392	142 (41)	130 (37)	0.035	101 (24)	222 (53)	0.097
Rectum	36 (8)	68 (16)		73 (17)	31 (7)		50 (14)	26 (8)		38 (9)	56 (17)	
BRAF												
wt	105 (24)	244 (56)	0.399	229 (52)	121 (28)	0.339	148 (43)	122 (36)	0.533	116 (28)	209 (51)	0.052
mut	23 (5)	67 (15)		63 (14)	26 (6)		43 (12)	30 (9)		22 (5)	67 (16)	
KRAS												
wt	79 (18)	178 (41)	0.316	181 (42)	76 (17)	0.049	124 (37)	72 (21)	0.001	86 (21)	159 (39)	0.250
mut	46 (11)	129 (30)		108 (25)	68 (16)		64 (19)	77 (23)		48 (12)	114 (28)	

Chi-square test or Mann–Whitney *U* test (*) was used for statistical analyses

*n* number of cases, *WHO PS* WHO performance status, *Alk phosph* alkaline phosphatase, *M* male, *F* female, *mut* mutant, *wt* wild type

### CAF-associated claudin-2 status predicts progression-free survival in the SPCRC cohort

**Fig. 2:**
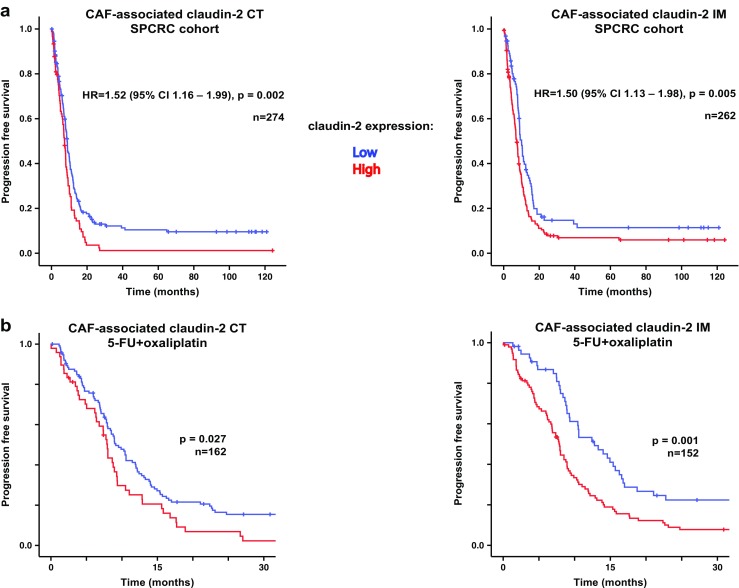
Associations between claudin-2 and progression-free survival (PFS). Kaplan–Meier graphs showing associations in the SPCRC cohort between PFS and claudin-2 expression in stromal cell/CAFs (**a**). Results from analyses of 5FU+oxaliplatin subgroup are shown in **b**. Results are shown separately for expression in central tumor (CT) (left panel) or invasive margin (IM) (right panel). HRs from Cox regression analyses, including confidence intervals, and *p* values are indicated for all analyses. Note, according to Bonferrony correction for the statistical significance, *p* value = 0.005 shall be considered as the threshold in the current illustration

No significant associations were detected between OS and claudin-2 expression in neither the malignant cells, nor the CAFs (data not shown). Furthermore, claudin-2 expression in the malignant cells was not associated with PFS (Supp. Fig. 6A). However, high expression of claudin-2 in CAFs in both CT and IM was significantly associated with shorter PFS (Fig. 2a) with median 8.8 and 7.4 months for low and high CAF-associated claudin-2 in CT, respectively (*p* = 0.002), and 10.1 and 7.4 months for low and high CAF-associated claudin-2 at IM, respectively (*p* = 0.005).

The univariate analyses were expanded to multivariate analyses, including performance status, alkaline phosphatase, BRAF, and KRAS mutation status. In these analyses, only the CAF-associated expression of claudin-2 in the IM acted as an independent predictor for PFS (Table 2).

**Table 2:** Stromal CAF-associated claudin-2 as a prognostic factor for PFS in multivariate analyses in patients with mCRC (SPCRC cohort)

	Univariate		Multivariate			
			CT, *n* = 253		IM, *n* = 237	
Variable	HR (95% CI)	*p* value	HR (95% CI)	*p* value	HR (95% CI)	*p* value
Stromal claudin-2 CT (high vs. low)	1.52 (1.16–1.99)	0. 002	1.28 (0.96–1.70)	0. 095	–	–
Stromal claudin-2 IM (high vs. low)	1.50 (1.13–1.98)	0. 005	–	–	1.43 (1.06–1.93)	0.018
Alkaline phosphatase (elevated vs. normal)	1.62 (1.23–2.14)	0.001	1.58 (1.19–2.10)	0.001	1.54 (1.14–2.08)	0.005
Performance status						
0 (reference)	1 (ref.)	0.176	1 (ref.)	0.002	1 (ref.)	0.003
(1 vs. 0)	1.22 (0.91–1.63)	0.176	1.20 (0.88–1.62)	0.251	1.27 (0.92–1.75)	0.142
(2–4 vs. 0)	1.39 (0.90–2.13)	0.134	2.08 (1.40–3.11)	< 0.001	2.03 (1.35–3.07)	0.001
BRAF (mut vs. wt)	1.65 (1.14–2.38)	0.008	1.73 (1.17–2.55)	0.006	1.63 (1.09–2.46)	0.019
KRAS (mut vs. wt)	1.25 (0.93–1.66)	0.135	1.38 (1.02–1.87)	0.036	1.33 (0.97–1.82)	0.082

*CT* tumor center, *IM* invasive margin, *HR* hazard ratio, *CI* confidence interval, *mut* mutant, *wt* wild type

### CAF-associated expression of claudin-2 at the invasive margin predicts response to oxaliplatin in the SPCRC cohorts

The survival analyses described above identified associations between claudin-2 status in CAFs with PFS, but not with OS. These findings prompted analyses exploring potential relationships between claudin-2 status and response to treatment. Additional PFS analyses were therefore performed to identify potential drug-specific associations between CAF-associated expression of claudin-2 and PFS. Depending on the treatment regimens used at the first-line treatment, three groups were identified: 5FU-alone group, 5FU+irinotecan, and 5FU+oxaliplatin group. The clinical characteristics of the patients from three treatment groups are shown in Supp. Table 2. Interestingly, the significant association between CAF claudin-2 and PFS, as determined by Cox regression analysis, was only detected in the 5FU+oxaliplatin group (*p* value = 0.027 and 0.001 for CT and IM CAF-associated claudin-2 expression, respectively, Fig. 2b and Supp Fig. 6B).

To expand these findings, multivariable analyses were performed on the treatment-defined subgroups (Table 3). High CAF-associated claudin-2 expression at IM then acted as a significant independent marker for shorter PFS in 5-FU+oxaliplatin group (*p* = 0.009). The expression in CT was not statistically significant (data not shown). Similar results were seen when 5-FU+oxaliplatin group was compared to merged 5FU-alone and 5FU+irinotecan group (data not shown).

**Table 3:** CAF-associated claudin-2 as a prognostic factor for progression-free survival in multivariate analyses in subgroups of the patients with received fluoropyrimidine alone, fluoropyrimidine with irinotecan, and those who received fluoropyrimidine with oxaliplatin for the first-line therapy in mCRC (SPCRC cohort)

	5-FU				5-FU+irinotecan				5-FU+oxaliplatin			
	Univariate		Multivariate, *n* = 48		Univariate		Multivariate, *n* = 41		Univariate		Multivariate, *n* = 144	
Variable	HR (95% CI)	*p* value	HR (95% CI)	*p* value	HR (95% CI)	*p* value	HR (95% CI)	*p* value	HR (95% CI)	*p* value	HR (95% CI)	*p* value
CAF-associated claudin-2 IM (high vs. low)	0.84 (0.57–1.52)	0.565	0.56 (0.27–1.13)	0.105	1.06 (0.55–2.03)	0.862	1.17 (0.54–2.54)	0.683	1.84 (1.27–2.67)	0.001	1.82 (1.23–2.71)	0.003
Alkaline Phosphatase (elevated vs. normal)	1.29 (0.82–2.02)	0.275	1.79 (0.86–3.72)	0.119	0.97 (0.60–1.56)	0.896	1.05 (0.49–2.25)	0.892	1.70 (1.29–2.23)	< 0.001	1.81 (1.21–2.71)	0.004
Performance status												
0 (reference)	1 (ref.)	0.261	1 (ref.)	0.054	1 (ref.)	0.207	1 (ref.)	0.456	1 (ref.)	0.002	1 (ref.)	0.573
0 (1 vs. 0)	1.27 (0.72–2.24)	0.407	1.62 (0.71–3.72)	0.252	1.10 (0.64–1.91)	0.731	0.79 (0.34–1.83)	0.584	1.16 (0.87–1.56)	0.316	1.15 (0.74–1.78)	0.540
0 (2–4 vs. 0)	1.67 (0.90–3.11)	0.104	3.58 (1.27–10.09)	0.016	1.99 (0.93–4.24)	0.076	1.79 (0.53–5.91)	0.349	2.08 (1.38–3.15)	< 0.001	1.38 (0.72–2.64)	0.333
BRAF (mut vs. wt)	1.02 (0.50–2.07)	0.957	2.57 (0.97–6.78)	0.067	2.70 (0.61–11.91)	0.190	3.13 (0.58–16.76)	0.183	1.32 (0.87–1.99)	0.187	1.37 (0.82–2.30)	0.227
KRAS (mut vs. wt)	1.45 (0.80–2.61)	0.221	1.95 (0.85–4.46)	0.115	1.20 (0.67–2.14)	0.546	1.30 (0.59–2.85)	0.519	0.94 (0.66–1.33)	0.718	1.24 (0.82–1.88)	0.314
Age (> 60 vs. ≤ 60)	1.20 (0.83–1.75)	0.339	1.46 (0.76–2.81)	0.252	1.44 (0.92–2.26)	0.114	0.96 (0.46–1.99)	0.908	0.75 (0.58–0.95)	0.019	0.72 (0.50–1.03)	0.069

*IM*, invasive margin, *HR* hazard ratio, *CI* confidence interval, *mut* mutant, *wt* wild type

These observations indicate that stromal expression of claudin-2 in the IM in primary CRC tissue predicts the response to oxaliplatin in first-line 5-FU-based treatment of mCRC.

### Stromal claudin-2 expression predicts survival also in the NORDIC-VII cohort of mCRC

Additional analyses were performed to investigate if the findings could be reproduced in an independent patient cohort. For this purpose, a NORDIC-VII study derived TMA was analyzed with regard to CAF-associated expression of claudin-2. All patients in the NORDIC-VII trial received combined 5-FU plus oxaliplatin therapy (see “Materials and methods”).

**Fig. 3:**
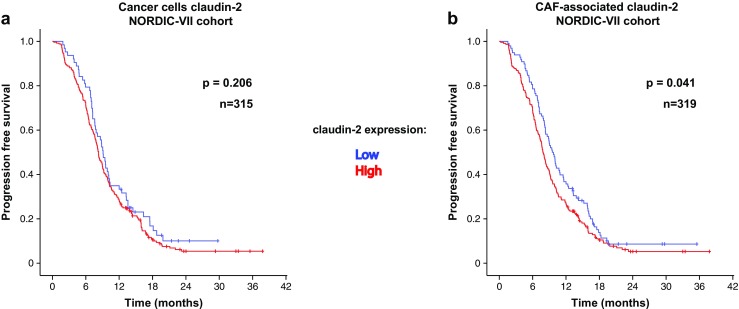
Associations between stromal/CAF-associated claudin-2 expression and PFS in the NORDIC-VII cohort. Kaplan–Meier graphs showing associations in the NORDIC-VII cohort between PFS and claudin-2 expression in cancer cells (**a**) or in stromal cell/CAFs (**b**)

In agreement with the findings from the SPCRC cohort, a statistically significant association between CAF-associated claudin-2 expression (but not cancer cell-associated claudin-2 expression) and PFS was detected (Fig. 3a, b).

This analysis provides independent evidence implying CAF-associated claudin-2 as a marker related to survival in mCRC treated with 5-FU+oxaliplatin. Notably, the treatment regimens of the NORDIC-VII population prevented analyses on oxaliplatin-specific survival associations CAF-associated claudin-2.

### Claudin-2 expression is concordant in primary tumor and in metastases

The analyses above on survival of mCRC were based on claudin-2 expression in primary tumors. This prompted analyses where the marker expression in primary tumor and patient-matched metastases was compared. These analyses were restricted to the subset of cases where matched primary tumor and metastatic tissue was available. As shown in Supp. Table.3, claudin-2 expression showed good concordance between primary and metastatic tissue in both cancer cells and CAFs.

## Discussion

Claudin-2 overexpression has been observed in CRC tissue. This is a first study to our knowledge, which reports a cell-type-specific analysis of the expression pattern of the protein in CRC.

We confirmed earlier described claudin-2 expression in cancer cells and described three distinct expression patterns. The intracellular localization patterns of the claudin-2 were not associated with the abundance of the protein. Notably, a survival association was detected for the unpolarized pattern. Possibly, this finding could be related to cell polarization/differentiation status of cells, and thus not causally linked to claudin-2-related biology.

The key findings of the present study are that claudin-2 is expressed in CAFs in primary tumor tissue of mCRC in a manner which is linked to KRAS mutation status and progression-free survival. Notably, the progression-free-survival association is restricted to the subset of patients treated with 5-FU+oxaliplatin.

CAF expression of claudin-2 in CRC has not been previously reported. However, other TJ proteins such as occludin and claudin-11 have been detected in CAFs of other tumor types [11]. Preliminary mechanistic studies have linked TJ protein expression in CAFs to increased migratory activity [11]. Further mechanistic studies on how claudin-2 expression affects CRC CAF phenotypes are warranted by the findings of the present study.

We report an association between oncogenic KRAS status and claudin-2 expression in malignant cells. Earlier studies have implied TGF-beta as an inducer of some TJ proteins in CAFs and epithelial cells [11, 15]. On another hand, some studies indicate that claudin-2 expression upregulates TGF-beta production in colonic epithelial cells and induce immune suppression [1]. Future studies should thus explore if the associations between oncogenic RAS and CAF-associated claudin-2 involves RAS-induced TGF-beta production.

The treatment-specific associations between claudin-2 and outcome are intriguing and merit further investigation. Experimental studies should explore if claudin-2 status of fibroblasts can regulate 5FU/oxaliplatin-sensitivity of cocultured CRC cells. Animal studies can also be performed where 5FU/oxaliplatin-sensitivity can be analyzed in tumor xenografts formed after coinjection of CRC cells and CAFs of defined claudin-2 status.

CAF subsets may have different impact on drug delivery by affecting the IFP. It has been shown before that increased fluid flow, caused by the differences between IFP in tumor and peritumoral regions can affect delivery of molecules in a manner that is related to their molecular weight [3, 16]. Molecular weights of both fluorouracil and oxaliplatin are comparable (0.130 and 0.397 kDa, respectively). Possibly, differential associations with macromolecules could affect delivery of the drugs and the IFP dependency. Future experimental studies on this topic are warranted.

Future studies should also consider the possibility that the oxaliplatin-treated cases represent a subgroup of CRC where claudin-2 expression is related to intrinsic tumor aggressiveness. In the SPCRC cohort, the oxaliplatin-receiving group differs from the fluoropyrimidine-alone-treated group by being younger and by displaying better WHO PS (Supp. Table 2). The CAF claudin-2 survival association remained in analyses performed in age- or PS-defined oxaliplatin-treated subgroups (data not shown) and in multivariate Cox regression models. Age or WHO PS is thus not explaining the treatment-specific CAF claudin-2 association with survival. The association seen in the Nordic VII study, where all patients received oxaliplatin, further strengthens our conclusion that claudin-2 expression is related to oxaliplatin treatment response.

Findings of the present study provide additional support for the notion that CAFs are clinically relevant regulators of drug sensitivity. As such, findings should stimulate to continued explorations of CAFs as sources of mechanistically relevant prognostic and response-predictive biomarkers.

## Electronic supplementary material

ESM 1 (DOCX 24361 kb)
