# Supplementary material for: Treatment-related survival associations of claudin-2 expression in fibroblasts of colorectal cancer
Source: Virchows Arch. 2017 Nov 13;472(3):395–405. doi: 10.1007/s00428-017-2263-3 (PMC5887004; doi:10.1007/s00428-017-2263-3)
Supplement: Supplementary file 1 — (DOCX 24361 kb) [file 428_2017_2263_MOESM1_ESM.docx]

***Supplementary material***

**Treatment-related survival associations of claudin-2 expression in fibroblasts of colorectal cancer**

Artur Mezheyeuski et al.

**Supplementary Materials and Methods**

**Patient population**

The SPCRC cohort is an unselected population diagnosed with non-resectable mCRC patients during 2003-2006 in three Scandinavian counties [[1](#_ENREF_1)]. In this cohort tissue microarray (TMA) could be produced in 462 (58%) of 798 initial cases; remaining patients had biopsies being too small, not containing tumour tissue or not available. Most patients received combination chemotherapy, which included a fluoropyrimidine and irinotecan or oxaliplatin.

The randomized NORDIC-VII study investigated the effects of combining cetuximab with a regimen of bolus 5-flourouracil (5-FU)/folinic acid (FA) and oxaliplatin ( FLOX) in first-line therapy of mCRC [[2](#_ENREF_2)]. Patients were randomly assigned to receive standard Nordic FLOX (arm A), cetuximab and FLOX (arm B), or cetuximab combined with intermittent FLOX (arm C). 571 patients were included between May 2005 and October 2007. Since there was no statistically significant difference in outcome between the treatment arms, the present study used the whole patient population across the different treatments in NORDIC-VII [[2](#_ENREF_2)].

**Tissue microarray (TMA)**

Tissue microarrays (TMAs) were made from formalin-fixed and paraffin-embedded tissue blocks of primary tumour. The SPCRC TMA contained multiple cores collected from tumour centre and invasive margin. For the current study, TMAs from 274 and 262 patients from the invasive margin and tumour centre, respectively, from the SPCRC cohort and from 315 patients of the NORDIC-VII cohort were available for immunohistochemistry. The main cause of the TMA core exclusion from the subsequent analyses was detachment of the tissue from the glass during the staining procedure.

In a number of TMA cores from the invasive margin the malignant cells were absent, which leads to decease the quantity of the cases applicable for cancer-cell-related scorings (n=214). Not all patients received first-line therapy in SPCRC cohort, thus the case number available for treatment sub-group analysis was lover (265 and 253 for TMA cores from tumour centre and invasive margin respectively)

**IHC procedures**

***Claudin-2 single staining.*** 4 µm thick TMA sections were subjected to antigen retrieval by boiling in pH 6.0 buffer after de-paraffinization and rehydration. Sections were thereafter incubated overnight with anti-claudin-2 mouse monoclonal antibody (Thermo Fisher Scientific Cat# 32-5600) at dilution 1:300 followed by 30 minutes incubation with amplification system (EnVision + System-HRP Dako A/S, EnVisionTM, Dako, CA, USA). Diaminobenzidine was used to develop the specific staining and haematoxylin as counterstain.

***Double staining procedures.*** Sections were de-paraffinized, rehydrated and subjected to antigen retrieval as described above. Sections were incubated overnight with antibodies against claudin-2 (Thermo Fisher Scientific Cat# 32-5600) at dilution 1:300. Thereafter, sections were incubated with polymer system (ImmPRESS™-AP Polymer Anti-mouse IgG MP-5402, Vector Laboratories, Burlingame, CA) for one hour at room temperature, and developed with Vector^®^ Blue AP Substrate Kit (SK-5300, Vector Laboratories, Burlingame, CA). After heating in decloaking chamber at 95 degrees C for 5 minutes, in pH 6.0 solution, sections were incubated overnight with pan-cytokeratin antibody (Clone AE1/AE3; Dako, Inc., Denmark) at dilution 1:300 or CD68 antibody (Clone PG-M1; Dako, Inc., Denmark) at dilution 1:100. Sections were then incubated with secondary antibodies for one hour at room temperature, and developed with Vector^®^ Red AP Substrate Kit (SK-5100, Vector Laboratories, Burlingame, CA).

***Evaluation of IHC****.* Stained slides were scanned with either the automated scanning system Aperio XT (Aperio Technologies, Inc.) and then viewed with the image viewer program ImageScope v. 11.1.2.752 (Aperio Technologies, Inc.) or scanned by a Vslide slide scanning microscope (Metasystems, Alltlussheim, Germany) using ×10 objective and RGB led illumination for color deconvolution and viewed with the program Metaviewer (Metasysetms, Alltlussheim, Germany).

The staining intensity on the digital slides was evaluated using a four-graded scale (negative (0), weak (1), moderate (2) or strong (3)). For survival analyses these scores were dichotomized to low (negative and weak) and high (moderate and strong) expression. In 48 TMA cores from the invasive margin in SPSRS the cancer cells were not observed or were represented by minor cell clusters. The claudin-2 expression in cancer cells were not evaluated in such cases, which leads to decrease in number of the cases from 262 to 214.

**ISH procedures**

RNAscope® 2.5 HD Reagent Kit-RED (Advanced Cell Diagnostics, Hayward, CA) was used to detect CLDN2 transcript. A custom-designed RNAscope probe targeting 489-1408 of NM_020384.3 was used to stain CRC tissue. 4 µm thick TMA sections were pre-treated by heating at 60°C for 60 minutes and then de-paraffinized and rehydrated. Staining procedure were performed according to the recommendation of the manufacturer and included pre-treatment with hydrogen peroxide for 10min and boiling with target retrieval solution for 15 min and protease digestion at 40ºC for 30 minutes. The hybridization with probe Hs-CLDN2 was performed for 2 hours at 40ºC. The signal was visualized with Fast RED and cell nuclei were counterstained with haematoxylin.

**Fibroblast isolation from tumour tissue**

The tissue samples were collected from 5 patients who had surgery for colon cancer in 2015 in Karolinska University Hospital Solna, Stockholm, Sweden. The patients were not treated with chemotherapy or radiation therapy before surgery. The tumour tissue samples were obtained after surgery, cut in small pieces and incubated in digestion media (Collagenase/Hyaluronidase (Stem cell Technologies, 10x stock), 1mg/ml Dispase (Stem Cell Technologies) and c=50ug/mlDNAse (Stem cell technologies) mixed in DMEM media without serum) at 37°C for 2-4 hours with occasional vortexing. Undigested pieces were removed.

Cells were collected by centrifugation for 10min at 1200rpm. Fibroblasts were separated from epithelial cells by quick trypsin (Hyclone) incubations, repeated over several passages.

The primary fibroblasts were cultured in Dulbecco's Modified Eagle Medium (DMEM) supplemented with 10% fetal calf serum, 100 units/ml penicillin, 100 units/ml streptomycin and 2 mM glutamine (all from Hyclone, GE Healthcare). Cells were kept at 37 °C in a humidified 5 % CO2 atmosphere.

**Immunofluorescence analyses**

For immunofluorescence (IF) analysis, cells were seeded onto 8-well chamber slides with glass bottom with 2 x 100000 cells per well. Cells were fixed with 4% formaldehyde for 15 minutes at room temperature followed by rinsing three times in PBS for five minutes each. Permeabilization was performed with ice-cold 100% methanol for 10 minutes at –20°C followed by washing in TBS-T (0.5% tween) for 5 minutes. Non-specific binding sites were blocked with 5% BSA in TBS-T for 1 hour, sections were incubated with the primary rabbit or mouse antibody to claudin-2 (Thermo Fisher Scientific Cat# 710221 or Thermo Fisher Scientific Cat# 32-5600) over night at 4°C. After three washing steps, slides were incubated with secondary antibody Goat anti-Rabbit IgG (H+L), Alexa Fluor® 488 at dilution 1:250 or Goat anti-Mouse IgG (H+L) Secondary Antibody, Alexa Fluor® 633 in TBS-T for 1 hour at room temperature. After washing in TBS-T for 1 hour, sections were incubated with second primary antibody to α-SMA (Clone 1A4; Dako, Inc., Denmark at dilution 1:300) or E-Cadherin ((24E10) Rabbit mAb, Cell Signaling Technology at dilution 1:300) over night at 4°C. The staining was visualized by the Alexa Fluor® 633 conjugate or Alexa Fluor® 488.

The tissue sample for IF was collected from a patient who had surgery for colon cancer in 2016 in Karolinska University Hospital Solna, Stockholm, Sweden. It was obtained after surgery, and frozen at -80°C. 10 µm thick sections were fixed with ice-cold methanol/acetone solution and incubated overnight with mouse antibody to claudin-2 (Thermo Fisher Scientific Cat# 710220) and visualized with Alexa Fluor® 488 as described above. Then CD163 mouse monoclonal antibody was added (NovocastraTM Liquid Mouse Monoclonal Antibody CD163, Product Code: NCL-L-CD163) at dilution 1:300 and visualized with Alexa Fluor® 633 as described above.

Slides were mounted using fluorescent mounting medium with diamidinophenolindole (DAPI) (Vectashield) to stain the DNA. The AxioVision Rel. 4.6 Software (Carl Zeiss) was used for visualization.

**Monocyte isolation and differentiation**

Human monocytes were isolated from heparinized blood, obtained from healthy donors (The Republic Research & Production Centre for Transfusiology and Medical Biotechnologies, Minsk, Belarus). Blood sample collection and its usage for scientific needs was approved by the local ethical committee. After sedimentation of the erythrocytes the blood plasma was centrifuged at 1500 rpm for 30 min with Histopaque (Sigma). The mononuclear fraction was collected and purified.

Mononuclear cells were incubated in RPMI 1640 (Lonza) supplemented with 100 U/ml penicillin, 100 μg/ml streptomycin and 2 mM l-glutamine. After 30 minutes of incubation in cell plates, the adherent cells (M0) were washed with phosphate buffered saline and cultured for 24h in RPMI 1640.

Monocytes were polarized to M2 by incubation with 40 ng/ml IL-4 (R&D Systems, Minneapolis, MN, USA) and/or 2 ng/ml M-CSF (PeproTech, Rocky Hill, NJ, USA) for 6 days.

After detachment in Versene solution and subsequent washing the cells were used to prepare cytospins slides by centrifuging onto glass microscope slides. These cytospin slides were dried, fixed with ice methanol (10 min) and acetone (2 min). Obtained cytospins were stained with antibodies to claudin-2 (Thermo Fisher Scientific Cat# 710221) and visualized by F(ab')2-Goat anti-Mouse IgG (H+L) Secondary Antibody, Alexa Fluor® 488 conjugate (Life Technologies). Images were obtained on automated Leica DM5000 B microscope using a Leica DFC420 C digital camera.

For quantitative evaluation of the expression of claudin-2, the ImageJ software (<http://rsb.info.nih.gov/ij>) was used. Arbitrary metrics were generated by multiplying the claudin-2-positive area and its expression intensity and normalization of that value to the number on cells, defined by DAPI.

**Statistical analyses**

Cox proportional hazards model was used to estimate statistical significance and relative hazards in univariate and multivariate settings. Goodman-Kruskal gamma test was used for the analyses of marker expression in different locations. Mann–Whitney U test and ANOVA tests were used for the analyses of associations between marker expression and clinical characteristics. All statistical tests were two-sided and *P* values <0.05 considered statistically significant. All statistical analyses were performed using SPSS V20 (SPSS Inc., Chicago, IL).

**References**

**[1] H. Sorbye, A. Dragomir, M. Sundstrom, P. Pfeiffer, U. Thunberg, M. Bergfors, K. Aasebo, G.E. Eide, F. Ponten, C. Qvortrup, B. Glimelius, High BRAF Mutation Frequency and Marked Survival Differences in Subgroups According to KRAS/BRAF Mutation Status and Tumor Tissue Availability in a Prospective Population-Based Metastatic Colorectal Cancer Cohort, PLoS One, 10 (2015) e0131046.**

**[2] K.M. Tveit, T. Guren, B. Glimelius, P. Pfeiffer, H. Sorbye, S. Pyrhonen, F. Sigurdsson, E. Kure, T. Ikdahl, E. Skovlund, T. Fokstuen, F. Hansen, E. Hofsli, E. Birkemeyer, A. Johnsson, H. Starkhammar, M.K. Yilmaz, N. Keldsen, A.B. Erdal, O. Dajani, O. Dahl, T. Christoffersen, Phase III trial of cetuximab with continuous or intermittent fluorouracil, leucovorin, and oxaliplatin (Nordic FLOX) versus FLOX alone in first-line treatment of metastatic colorectal cancer: the NORDIC-VII study, J Clin Oncol, 30 (2012) 1755-1762.**

**Supp. Table 1.**

Associations between claudin-2 expression in different tumour regions and in different cell types (SPCRC cohort)

| **Variable** | **Cancer cells claudin-2 IM** | | | **CAF claudin-2 CT** | | | **CAF claudin-2 IM** | | |
| --- | --- | --- | --- | --- | --- | --- | --- | --- | --- |
|  | n | g | p-value | n | g | p-value | n | g | p-value |
| **Cancer cells claudin-2 CT** | 344 | 0.63 | <0.001 | 444 | 0.28 | <0.001 | 410 | 0.19 | 0.002 |
| **CAF claudin-2 IM** | 349 | 0.24 | 0.001 | 411 | 0.37 | <0.001 |  |  |  |
| **CAF claudin-2 CT** | 343 | 0.06 | 0.47 |  |  |  |  |  |  |

Goodman-Kruskal gamma Test was used for statistical analyses

**Supp. Table 2.**

Associations between treatment groups and clinicopathological parameters in patients from the SPCRC cohort of metastatic colorectal cancer

|  | Treatment groups | | |  |
| --- | --- | --- | --- | --- |
|  | 5FU | 5FU+irinotecan | 5-FU  +oxaliplatin | p value |
|  | n, (percent) | n, (percent) | n, (percent) |  |
| Median age (range) | 77  (53-88) | 62  (22-74) | 61  (26-81) | <0.001* |
| WHO PS |  |  |  |  |
| 0 | 21 (21) | 57 (65) | 145 (55) |  |
| 1 | 47 (48) | 21 (24) | 83 (32) | <0.001 |
| 2-4 | 31 (31) | 10 (11) | 34 (13) |  |
| Alk phosph |  |  |  |  |
| normal | 43 (47) | 30 (36) | 115 (46) | 0.223 |
| elevated | 48 (53) | 54 (64) | 137 (54) |  |
| Gender |  |  |  |  |
| M | 48 (49) | 51 (58) | 139 (53) | 0.432 |
| F | 51 (51) | 37 (42) | 123 (47) |  |
| Location |  |  |  |  |
| colon | 68 (70) | 47 (54) | 171 (66) | 0.056 |
| rectum | 29 (30) | 40 (46) | 88 (34) |  |
| BRAF |  |  |  |  |
| wt | 43 (78) | 47 (94) | 131 (79) | 0.046 |
| mut | 12 (22) | 3 (6) | 34 (21) |  |
| KRAS |  |  |  |  |
| wt | 32 (59) | 26 (51) | 101 (62) | 0.379 |
| mut | 22 (41) | 25 (49) | 62 (38) |  |

Abbreviations: n, number of cases; WHO PS, WHO performance status, Alk phosph, alkaline phosphatase; M, male; F, female; mut, mutant; wt, wild type.

Chi-Square Test, or Kruskal-Wallis Test (*) was used for statistical analysis

**Supp. Table 3.**

Associations between claudin-2 expression in primary tumour and metastatic tissue (NORDIC-VII cohort)

| **Variable** | **Cancer cells claudin-2**  **n=48** | | **CAF claudin-2**  **n=51** | |
| --- | --- | --- | --- | --- |
|  | g | p-value | g | p-value |
| Primary tumor and metastatic tissue expression | 0.53 | 0.003 | 0.44 | 0.006 |

Goodman-Kruskal gamma Test was used for statistical analyses

**Supp. Figure 1.** ***Claudin-2 expression in human colorectal cancer tissue.*** Micro-photographs illustrating cellular distribution of claudin-2 expression. (**A (a-c)**). Examples of different expression pattern of claudin-2 in cancer cells with basal (a), supranuclear (b) and un-polarized (c) expression. (**B**) Expression of claudin-2 in endothelial cells. Double staining with claudin-2 (blue) and CD34 (red) identifying double-positive cells. (**C**) Expression of the claudin-2 in macrophages. Double staining with claudin-2 (blue) and CD68 (red) identifying double-positive cells marked with arrow points.

**Supp. Figure 2. *Associations between claudin-2 expression pattern in cancer cells and OS.*** Kaplan-Meier graphs showing associations in the SPCRC cohort between the three distinct claudin-2 expression pattern in cancer cells and OS.

**Supp Figure 3.** ***Claudin-2 expression macrophages.*** Micro-photographs illustrating macrophage-associated claudin-2 expression. Pictures show double staining with claudin-2 (A and B blue; C red) and M2 macrophage marker CD163 (A and B red; C green). (**A**) Representative image of non-cancerous colonic mucosa. A fraction of claudin-2+/CD163+ cells localized restricted to the subepithelial region. (**B1**) Tumour invasive margin region characterized by big fraction of claudin-2+/CD163+ cells. (**B1**) Peritumoral tissue, characterized by low fraction of claudin-2+/CD163+ cells.

(**C**) Immunofluorescence image, illustrating the double positivity of the cells with macrophage morphology to CD163 and claudin-2. Picture is taken from tumour tissue.

**Supp Figure 4.** ***Claudin-2 expression in differentiated macrophages.*** Micro-photographs illustrating expression of claudin-2 during human monocyte-macrophage differentiation *in vitro*. (**A-D**) Representative images of mononuclear cells differentiated under stimulation with IL-4, M-CSF or IL-4+M-CSF. (**E**) A plot, showing the levels of expression of claudin-2 in mononuclear cells under stimulation with IL-4, M-CSF or IL-4+M-CSF. Arbitrary units represent metrics, generated by multiplying the claudin-2-positive area and its expression intensity and normalization of that value to the number on cells, defined by DAPI.

**Supp Figure 5.** ***Claudin-2 expression in fibroblasts, co-cultured with caco-2 cells (A).*** Note expression of claudin-2 (green) in cells (shown by white arrowheads), negative for E-cadherin staining (red). Two representative areas are shown. ***Illustrative examples of claudin-2 protein (left panel, IHC) and mRNA (middle and right panel, ISH) localisation in human tissue (B).*** Note expression of claudin-2 within a representative stroma-high sample (B1) is detected in fibroblasts (arrows) and macrophage-like cells (arrowheads). In a representative stroma-low case (B2) claudin-2 is detected almost exclusively in epithelial cells. Macrophage-like cell as well as non-malignant colon epithelium, also show positivity for claudin-2 in peritumoral non-cancerous mucosa (B3). Note the accordance between claudind-2 protein and mRNA expression.

**Supp Figure 6.** ***Associations between claudin-2 and progression-free survival (PFS).*** Kaplan-Meier graphs showing associations in the SPCRC cohort between PFS and claudin-2 expression in cancer cells (A). Results from analyses of treatment-defined subgroups are shown in (B). Results are shown separately for expression in central tumour (CT) (left panels) or invasive margin (IM) (right panels). HRs from Cox-regression analyses, including confidence intervals, and p-values are indicated for all analyses. Note, according to Bonferrony correction for the statistical significance, P value =0.005 shell be considered as the threshold in the current illustration.
